# Effects of Music Volume Preference on Endurance Exercise Performance

**DOI:** 10.3390/jfmk7020035

**Published:** 2022-04-27

**Authors:** Kylie M. Nixon, Mckenzie G. Parker, Carson C. Elwell, Anna L. Pemberton, Rebecca R. Rogers, Christopher G. Ballmann

**Affiliations:** Department of Kinesiology, Samford University, Birmingham, AL 35229, USA; knixon@samford.edu (K.M.N.); mparke10@samford.edu (M.G.P.); celwell@samford.edu (C.C.E.); apembert@samford.edu (A.L.P.); rrogers1@samford.edu (R.R.R.)

**Keywords:** motivation, rowing, preferred, non-preferred, rate of perceived exertion

## Abstract

The purpose of this study was to examine the effects of preferred versus non-preferred music volume on relative power output, trial time to completion (TTC), heart rate (HR), rate of perceived exertion (RPE), and motivation during endurance rowing exercise. Physically active females (age 18–25) volunteered to participate. In a crossover counterbalanced design, participants completed two trials: non-preferred (NPV) and preferred (PV) music volume. Participants began with a rowing warm-up at 50% of HRmax for 5 min. Following this, participants completed a 2000 m rowing time trial as quickly as possible. Relative power output, HR, and RPE were documented each minute during the exercise bout. TTC and motivation levels were documented at the cessation of exercise. Results showed that there were no significant differences between NPV and PV for relative power output (*p* = 0.287; d = 0.17), TTC (*p* = 0.816; d = 0.01), and HR (*p* = 0.956; d = 0.08). However, RPE was significantly lower (*p* = 0.002; d = 0.86) and motivation was significantly higher (*p* < 0.001; d = 2.14) during the PV condition versus NPV. Findings suggest that while PV does not impart performance-enhancing effects during endurance exercise compared to NPV, it may improve psychological responses related to intensity and effort which could have important implications for enduring intense exercise and training.

## 1. Introduction

The effects of music on exercise performance have been prodigiously investigated resulting in a plethora of evidence supporting ergogenic benefit [1,2]. Indeed, music has been shown to unequivocally provide ergogenic aid in endurance [3,4], anaerobic sprint [5,6], and resistance-based exercise [7,8]. Recent evidence has shown that individual preference related to music may be a key mediator to performance enhancement [2]. Specifically, preferred music genre has been well established to improve psychophysiological responses to exercise and performance [2]. Musical compositions are multi-faceted and various components including genre, tempo, lyrical content, and volume/intensity may influence perceptions of music. However, few investigations outside of music genre have been conducted leaving the influence of preference of other musical components on exercise performance relatively unknown.

Preferred music genre has been shown to improve exercise performance compared to non-preferred in multiple modes of exercise [2,3,7,8,9,10]. For example, Bartolomei et al. showed that preferred music increased repetition volume and strength-endurance during bench press exercise [8]. Furthermore, our lab has shown that listening to a preferred music genre during a warm-up results in higher power output and faster performance time while completing a 2000-m rowing trial [4]. Fundamental to most musical preference and exercise investigations are marked increases in motivation to exercise [2,9,11,12,13]. Since increases in motivation may lead to greater effort and drive [14], underlying ergogenic benefits may be dependent on motivational improvement. Whether or not other components of musical composition influence motivation similarly is unknown necessitating further study.

Varying music volume has been shown to elicit distinct physiological and psychological changes [15]. Listening to intense music has been shown to increase HR, respiratory rate, and decrease HR variability suggesting increases in sympathetic output [16]. Loud music has also been documented to increase psychological arousal and alertness [17]. Pertaining to exercise, loud/intense music has been shown to produce greater ergogenic effects compared to soft/sedative music and no music [18,19]. Edworthy et al. showed that loud/fast music resulted in faster running speeds and HR compared to soft and no music [18]. Supporting this further, Karageorghis et al. showed that loud/fast music resulted in improved grip strength [19]. Lower grip strength was also observed with soft/fast music. However, overstimulation can occur at excess volumes which may, in turn, hinder performance [15]. Anecdotally, music played at gyms, locker rooms, and sporting events is played at louder volumes. Identifying whether performance is hindered or optimized if individuals prefer different music volume levels may help to optimize performance in these settings.

To date, we are only aware of one investigation which has systematically examined music volume preference and exercise. Hutchison et al. showed that when participants were free to choose music volume level during a graded exercise test, they tended to choose louder music volumes as intensity increased [20]. Furthermore, perceived usefulness of music towards performance during exercise was highest at the onset of fatigue and ventilatory threshold [20]. These intriguing findings highlight the importance of individual music volume preference and suggest that it may be intimately linked to perceived ergogenic benefit. However, no studies to date have investigated how non-preferred music volume may influence performance compared to preferred levels. The purpose of this study was to elucidate the effects of preferred and non-preferred music volume on endurance exercise performance. We hypothesized that preferred music volume would result in higher power output, HR, motivation to exercise, and lower rate of perceived exertion (RPE) during a 2000-m rowing time trial.

## 2. Materials and Methods

### 2.1. Study Design

In a counterbalanced crossover manner, female participants completed 2 separate exercise sessions each with a different music volume condition: non-preferred music volume (NPV) and preferred music volume (PV). Following a standardized warm-up, participants listened to a song of their choosing at their self-reported NPV or PV as they completed a 2000-m endurance rowing time trial. Relative power output, HR, and RPE were recorded throughout the exercise. Time to completion (TTC) and motivation to exercise were recorded immediately following the cessation of exercise. Each visit was separated by a minimum of 48 h. Direct comparisons between NPV and PV were made for each outcome. Prior to any data collection, verbal and written informed consent was obtained from each participant. All experimental procedures were conducted in accordance with the Declaration of Helsinki and approved by the Samford University Institutional Review Board (EXPD-HP-20-F-1; August 2021).

### 2.2. Participants

Adequate sample size was determined using an a priori power analysis with G*power statistical software (G*power V 3.1.9.4). Our lab has shown repeatedly that music preference modulates motivation as a key mechanism for performance improvements [4,9,10,11,12,13]. A previous investigation from our group using an identical exercise protocol showed increases in motivation with preferred music genre preference with an estimated effect size of d = 1.45 [4]. To calculate the sample size needed, the following parameters were used: test = t-test (matched pairs), d = 1.45, α = 0.05, 1-β = 0.8. This equated to a minimum sample size of n = 6. In order to have comparable sample sizes to previous investigations [2,4,9,10,11,12,13], 12 physically active females (21.0 y ± 0.9, 54.0 kg ± 5.5, 165.3 cm ± 6.5) were recruited to participate. To be considered physically active, participants had to report attainment of at least 150 min/wk of moderate intensity exercise [21]. Other inclusion criteria included being free from orthopedic injury in the past six months prior to participation and no self-reported diagnosis of hearing loss. Participants also completed a physical activity readiness questionnaire (PAR-Q) to screen for safety of exercise which was required prior to participation. Participants were asked to refrain from vigorous activity 24 h prior and alcohol, caffeine, and nicotine 12 h prior to testing [4,22]. Participants were unaware of any experimental hypotheses.

### 2.3. Music, Volume Preference, and Familiarization

Upon the arrival at the first session, participants were instructed to pick any song of their liking so long as the tempo was ≥120 beats∙min^−1^ (bpm) as this has been suggested as the minimum tempo required to be considered stimulative [4,23]. Participants were then asked to rank 3 volume/decibel (dB) level choices from most preferred to least preferred for when they exercise: loud (85 ± 5 dB; small concert/loud singing), moderate (75 ± 5 dB; busy restaurant), and soft (65 ± 5 dB; normal conversation) [24]. Both the decibel level and reference example were presented to participants when choosing and ranges were chosen based on typical noise levels experienced in daily life and safety for hearing [24,25,26]. The highest-ranked volume level was used for the PV condition and the lowest-ranked for the NPV. Music was played over a 12-inch external speaker (Acoustic Amplification, Los Angeles, CA, USA) and volume was measured using a digital decibel meter (TACKLIFE, Levittown, NY, USA). The chosen song was played on repeat to ensure that music played the entirety of the exercise bout. Prior to beginning any exercise, participants were familiarized with the rowing ergometer (Concept2, Morrisville, VT, USA) as previously described by our lab [4]. Briefly, participants were shown correct form by a researcher and then given 3 rowing attempts to ensure proper execution. If necessary, form was corrected.

### 2.4. Procedures

All rowing exercise and testing procedures were followed as previously described by Karow et al. [4]. Participant height and weight were measured and then participants were outfitted with a chest-strap HR monitor (Polar Electro, Lake Success, NY, USA). For the warm-up, participants rowed for 5-min once they achieved 50% of their age-predicted HRmax. Following the warm-up, music was immediately started, and participants rowed 2000-m as quickly as possible. At every minute, HR, RPE (1–10 scale), and power output were documented then averaged for analysis. Time to completion and motivation to exercise were documented at the end of the exercise. For motivation, participants were asked to mark how motivated they felt to exercise on a 100 mm line ranging from “no motivation” to “extremely motivated” [4,10,11]. Participants were blinded to power output, time, HR, and distance throughout the entirety of the exercise bout.

### 2.5. Statistical Analysis

All analytics were completed using Jamovi software (Version 0.9; Sydney, Australia). To confirm normality of data, testing using the Shapiro–Wilk method was completed. Means from each condition were compared using a pairwise t-test. Effect sizes between means were calculated via Cohen’s d (d) and interpreted as: 0.2—small; 0.5—moderate; 0.8—large [27,28]. All data are presented as mean ± standard deviation (SD). Significance was set at *p* ≤ 0.05 a priori.

## 3. Results

**Figure 1:**
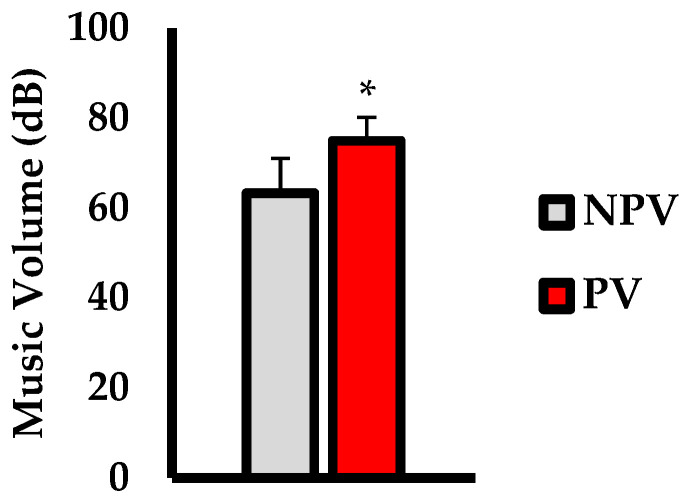
Average music volume chosen in decibels (dB) between non-preferred volume (NPV) and preferred volume (PV) conditions. Data are presented as mean ± SD. * indicates significantly different than NPV (*p* < 0.05).

**Figure 2:**
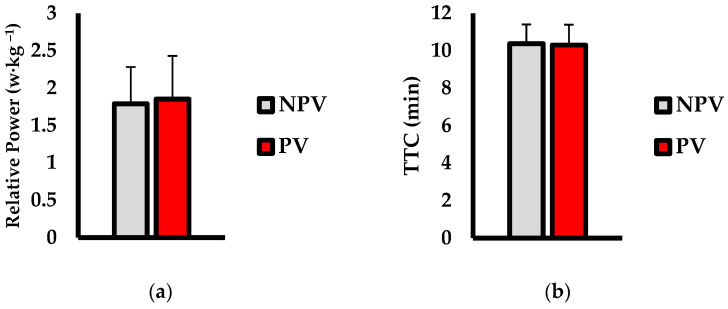
Comparisons of (**a**) relative power output (w·kg^−1^), (**b**) time to completion (TTC; min) between non-preferred volume (NPV) and preferred volume (PV) conditions. Data are presented as mean ± SD.

Average music volume chosen (dB) is shown in Figure 1. Music volume was significantly higher in the PV condition versus NPV (*p* = 0.003; d = 1.49). Performance outcomes are shown in Figure 2. For relative power output (w·kg^−1^; Figure 2a), there was no significant difference over the time trial between PV and NPV (*p* = 0.287; d = 0.17). Analysis of TTC (min; Figure 2b) showed no significant difference between conditions (*p* = 0.816; d = 0.01).

**Figure 3:**
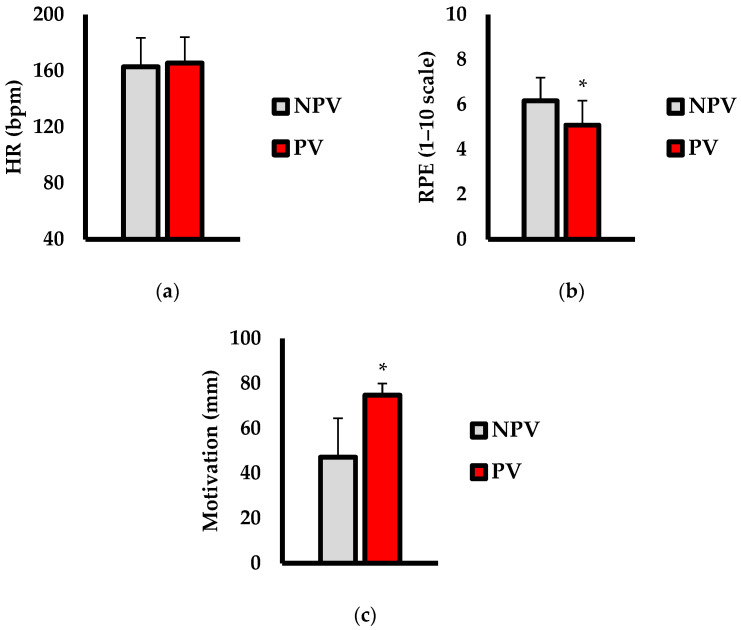
Comparisons of (**a**) heart rate (HR; bpm), (**b**) rate of perceived exertion (RPE; 1–10 scale), and (**c**) motivation to exercise (mm) between non-preferred volume (NPV) and preferred volume (PV) conditions. Data are presented as mean ± SD. * indicates significantly different than NPV (*p* < 0.05).

Analysis of psychophysiological outcomes are shown in Figure 3. No differences in HR (bpm; Figure 3a) were observed (*p* = 0.956; d = 0.08). For RPE (1–10 scale; Figure 3b), PV resulted in significantly lower ratings compared to NPV (*p* = 0.002; d = 0.86). Furthermore, motivation to exercise (mm; Figure 3c) was significantly higher (*p* < 0.001; d = 2.14) during the PV condition versus NPV.

## 4. Discussion

The ergogenic effects of listening to music have been well described [1]. Moreover, music genre preference has been repeatedly reported to determine the efficacy of performance enhancement [2]. Louder volumes/intensities of music have also been shown to result in superior benefits on performance compared to softer alternatives [6,17]. While a previous investigation has shown that participants preferred louder music during graded exercise [20], no studies to date have investigated whether PV or NPV differentially influence performance. Findings from the current study showed that participants preferred loud volumes during exercise and no changes in performance were seen regardless of volume. However, PV resulted in lower RPE and higher motivation to exercise versus NPV. While mechanistic determinations of psychophysiological changes with PV remain largely unknown, these findings emphasize the importance of consideration of music volume in order to optimize responses to exercise.

Participants in the present study preferred their music significantly louder compared to their non-preferred volume. This is in support of previous studies showing that adolescents and college students may prefer loud volumes while listening to music [29]. Despite this, no differences in performance between PV and NPV conditions were apparent. Lack of changes in power output and time trial performance are in opposition to previous genre preference studies [2,4,9,13]. For example, a previous study from our lab showed that listening to preferred music prior to a rowing time trial enhanced performance compared to no music [4]. Since the same song was used for both conditions, the standardization of genre may have undermined potential ergogenic benefits of the music. Indeed, varying music genres may affect emotional and psychological responses differently such as valence and arousal [30]. Emotional responses have been linked to differences in exercise performance and standardization of genre may have removed alterations of emotional state during the rowing bout [31]. Furthermore, lack of performance improvements may be due to tempo standardization since the identical song was played each time. Previous evidence has shown that pacing to stimulating music increases exercise efficiency during endurance exercise [32]. The identical tempo between conditions may have caused an identical synchronization effect resulting in no differences in performance. Supporting this, evidence from our lab has shown that tempo-matched asynchronous music of a preferred genre did not alter sprint performance compared to non-preferred despite improvements in psychophysiological measures [11]. As with music in general, the manner in which music preference influences performance is complex whereby mediators (i.e., genre, tempo, volume, etc.) are likely to be interdependent and work in a consortium to form optimal exercise responses. The exact contribution and interaction of each of these mediators to exercise performance remains unclear and more comprehensive study directly comparing each is warranted.

Changes in motivation and RPE have been repeatedly shown to be favorably altered by preferred music genre [2,3,9,10,11,12,13]. Current findings support this, showing that PV increased motivation and decreased RPE. However, nearly all prior studies standardized volume across all trials. Previous evidence has shown that on average, individuals choose louder music volume during graded exercise proportional to exercise intensity [20]. Furthermore, perceived usefulness of the music was higher with chosen volume. Collective findings may be due in part to louder volumes inducing a greater dissociation effect. Indeed, dissociation, as measured through RPE, has been suggested as a primary mechanism of the benefit of music on performance [1,2]. Larger amounts of dissociation may serve to distract exercisers from discomfort or fatigue during high-intensity exercise. Since loud music has been well-described to exacerbate attention diversion in various contexts [33], the louder volume of the PV may have elicited greater dissociation resulting in lower RPE. Furthermore, PV resulted in greater motivation to exercise which also bolsters previous work on music genre preference [2,4,9,10,11,12,13]. Loud sounds have been shown to increase sympathetic (i.e., fight or flight) and central nervous system drive which may increase alertness and arousal [34,35]. Indeed, higher perceived loudness has been reported to alter motor cortex activity and control [36]. Although purely speculative, PV may have led to a more optimal amount of neural stimulation and arousal, thereby increasing the feeling of motivation. However, current findings of no changes in HR, although trending, do not fully support this. No research exists examining physiological changes with varying music volume preference, which will be necessary to identify specific mechanisms underlying increases in motivation. While changing these variables did not lead to improvements in performance, alterations in RPE and motivation may increase acute exercise volume (i.e., amount of exercise performed) or possibly adherence to exercise over time. For example, RPE has been shown to be a predictor of fatigue and time to exhaustion during exercise in multiple contexts [37,38]. Thus, improvements in psychophysiological responses to high-intensity exercise with PV may result in adaptive responses over time, but future studies with longitudinal approaches are warranted before making conclusions.

The current study provides novel findings of how music volume preference influences exercise performance. However, limitations still exist. Firstly, the volume of the preferred volume condition was significantly higher than the non-preferred. Therefore, it is not possible to delineate whether current findings were due solely to volume preference or merely the higher music intensity. It is worth noting that music volume preference was still heterogenous with ~50% of participants choosing moderate volume as PV and ~17% choosing loud as NPV. Thus, it is likely that absolute music intensity and preference are interconnected with each other and may need to be considered synergistically for exercise responses. Also, a “no music” control was not included in the current study design which has been widely used in music preference research previously [2]. This was due to the already wide array of studies showing loud, fast, or preferred music to be ergogenic compared to no music [2,9,11,12,13,18,20]. Therefore, the reader is cautioned in their interpretation that only differences in volume preference, not the presence of music, were compared. The current sample only utilized young females which may not be representative of the general population, especially since females may react to music differently during exercise than male counterparts [39]. But, females are drastically understudied in these contexts which makes more intentional study of this population necessary to exhaust knowledge in this area [40,41]. Lastly, time of day was not strictly controlled for although drastic differences in times were avoided. We cannot rule out some influence of diurnal fluctuations in performance. Despite these limitations, current data provide advancement in the knowledge of music preference and exercise performance while highlighting the need for more studies into how different aspects of music may be preferred and how they may influence performance differently.

## 5. Conclusions

In conclusion, current findings showed that PV did not result in superior endurance exercise performance compared to NPV. However, PV resulted in higher motivation and lower RPE. These data suggest that music volume preference may impart a greater influence on psychophysiological outcomes rather than performance. From a practical standpoint, music played in communal settings (i.e., gyms, locker rooms, sporting events, etc.) is often played at varying volumes whether that be soft or loud. Present data suggest that individuals’ motivation and/or perceived fatigue in those settings, who do not prefer the music volume may suffer. Thus, individuals looking to improve motivation and lower perceptions of exertion may consider using headphones or other methods of ensuring that control of music volume is to their liking. This may ultimately improve training and sport outcomes but further research is needed to confirm this possibility.

## Data Availability

All data are freely available within this manuscript.
